# New Insights into the Binding and Catalytic Mechanisms of *Bacillus thuringiensis* Lactonase: Insights into *B. thuringiensis* AiiA Mechanism

**DOI:** 10.1371/journal.pone.0075395

**Published:** 2013-09-18

**Authors:** Marc N. Charendoff, Halie P. Shah, James M. Briggs

**Affiliations:** Department of Biology and Biochemistry, University of Houston, Houston, Texas, United States of America; Russian Academy of Sciences, Institute for Biological Instrumentation, Russian Federation

## Abstract

The lactonase enzyme (AiiA) produced by *Bacillus thuringiensis* serves to degrade autoinducer-1 (AI-1) signaling molecules in what is an evolved mechanism by which to compete with other bacteria. Bioassays have been previously performed to determine whether the AI-1 aliphatic tail lengths have any effect on AiiA’s bioactivity, however, data to date are conflicting. Additionally, specific residue contributions to the catalytic activity of AiiA provide for some interesting questions. For example, it has been proposed that Y194 serves to provide an oxyanion hole to AI-1 which is curious given the fact the substrate spans two Zn(2+) ions. These ions might conceivably provide enough charge to promote both ligand stability and the carbonyl activation necessary to drive a nucleophilic attack. To investigate these questions, multiple molecular dynamics simulations were performed across a family of seven acylated homoserine lactones (AHL) along with their associated intermediate and product states. Distance analyses and interaction energy analyses were performed to investigate current bioassay data. Our simulations are consistent with experimental studies showing that AiiA degrades AHLs in a tail length independent manner. However, the presence of the tail is required for activity. Also, the putative oxyanion hole function of Y194 toward the substrate is not observed in any of the reactant or product state simulation trajectories, but does seem to show efficacy in stabilizing the intermediate state. Last, we argue through ionization state analyses, that the proton shuttling necessary for catalytic activity might be mediated by both water and substrate-based intra-molecular proton transfer. Based on this argument, an alternate catalytic mechanism is proposed.

## Introduction

Quorum signaling/sensing (QS) is an intercellular communication mechanism where through the use of chemical signals organisms can detect their local population and change their gene expression so as to competitively optimize their behavior in their local environment. Both Gram-positive and Gram-negative bacteria display this phenomenon through the utilization of different types of diffusible chemical species. Gram-positive bacteria employ signaling molecules that are comprised of small peptides whereas Gram-negative bacteria largely use families of acylated homoserine lactones (autoinducer-1, *V* *. fischeri* ) [1]. Both Gram-positive and Gram-negative bacteria also synthesize families of ribose-like molecules that allow for interspecies communication known as autoinducer-2 ( *V* *. fischeri* ) [1]. These signaling molecules are synthesized by enzymes within bacteria, after which they diffuse into the extracellular environment. As the concentration of signaling molecules in the environment increases beyond a threshold level (the “quorum” concentration), diffusion of the signaling molecules back into the bacteria promotes a change in gene expression mediated by quorum signal-bound transcription factors. The resulting alteration in gene expression is not insignificant as organisms like *Pseudomonas aeruginosa* have proteome expression levels that have been observed to change by as much as 50% from its planktonic state to its full-fledged, mature biofilm state [2]. This results in phenotypic changes including production of exopolysaccharides and biofilm formation concomitant with changes in metabolism and pathogenic toxin production. Indeed, not only has biofilm production been suggested as a means of facilitating antibiotic resistance, but genes under the influence of QS molecules are also known to code for antibiotic efflux pumps that further protect the bacterium from external challenges [3]. In addition, there is also evidence that quorum signaling molecules themselves can produce an immunomodulatory effect in the host to the benefit of the guest organism [4]. Clearly, these phenotypical expressions are undesired from many different standpoints and comprise challenges in many different fields from medicine to engineering and public health.

*Pseudomonas aeruginosa* is a Gram-negative bacterium that has a well-studied quorum sensing system, some of whose signaling molecules consist of acylated homoserine lactones (AHLs). These molecules consist of a homoserine lactone head connected via an amide linkage to a tail with lengths of 4 to 16 carbons. Additionally, they sometimes contain hydroxyl or carbonyl groups at the third carbon atom [5]. There are two AHL-dependent quorum sensing systems in *P. aeruginosa*, las and rh1 that each contain homologs of the LuxR (quorum signal receptor) and LuxI (quorum signal synthase) proteins. The las and rh1 systems direct quorum sensing mediated by N-(3-oxododecanoyl)-L-homoserine lactone and N-butyryl-L-homoserine lactone, respectively [6]. The las and rh1 operationally comprise a layered system whereupon the LasR-autoinducer complex induces rh1R expression. The LasI autoinducer in turn interferes with binding of Rh1I-autoinducer to Rh1R, presumably to ensure sequential activation of the las and rh1 cascades [7]. The end result of these cascades is the expression of many different genes including the las-promoted lasA, a protease; lasB, an elastase; toxA, or ExotoxinA; and aprA, an alkaline phosphatase. Additional genes expressed by the rh1 system are rpoS, a stationary phase sigma factor; rhlAB, a rhamnosyltransferase involved in synthesis of the biosurfactant/hemolysin rhamnolipid; and lecA which encodes a cytotoxic lectin [7]. The LasI and Rh1I synthases are, of course, also produced in these autoinduction cycles. In addition to LasR and Rh1R, *P. aeruginosa* also possesses a third orphan receptor, QscR that lacks a cognate synthase. This receptor senses N-(3-oxododecanoyl)-L-homoserine lactone and serves to control its own as well as other QS genes.

The existence of enzymes whose purpose is to degrade quorum signaling molecules has been known for at least the last decade. These quorum quenching enzymes exist in prokaryotes and eukaryotes to facilitate prokaryote-prokaryote and prokaryote-eukaryote interactions. In some cases, quorum signaling bacteria themselves can produce quorum quenching enzymes that target their own signal molecules. In one study, quorum signal lactonase activity was observed in human airway epithelial cells upon testing transgenic expression of the AiiA gene in cell lines. The lactonase activity was conferred to the paraoxonase (PON) gene that displays activity toward over 30 different non-AHL type lactones. Later the same PON enzymes were found to also exhibit hydrolytic activity in other capacities, among which were nerve agent detoxification, and drug metabolism [8]. It has been demonstrated that a Gram-positive soil-dwelling bacterium, *Bacillus thuringiensis*, expresses a lactonase enzyme (AiiA) that targets and destroys the AHLs of *P. aeruginosa* through hydrolysis of the lactone ring. The enzyme has been subject to study and is of interest as its resultant effect is to disrupt *P. aeruginosa*’s (and that of other Gram-negative bacteria) ability to communicate, thus, stemming undesired phenotype expression. Some understanding of the enzyme’s mechanism of action has been obtained, but other functions have yet to be fully elucidated.

The *B. thuringiensis* AiiA enzyme is a 250-residue metalloprotease containing two zinc ions in close proximity. Sequence analysis reveals a conserved HXHXDH motif that is shared with the metallo-β-lactamase superfamily of proteins. The zinc ions are identified by the coordinating ligands of the protein, namely, H104, H169, H106, and H235, H109, and D108 for Zn1 and Zn2, respectively. D191 serves to ligate both Zn1 and Zn2. Crystal structure determinations and quantum mechanical studies have led to the suggestion that the lactone head adopts a bridging position across the zinc ions by orienting its ether and carbonyl oxygens toward Zn1 and Zn2, respectively. Crystal structure studies have elucidated an oxygen density that is consistent with a hydroxide ion or water molecule that also bridges the zinc atoms [10]. Given that metal associated water has a pKa of approximately 7.5, we reasoned that the oxygen density was most likely a hydroxide anion [9]. Kim et al. [7] and Liu et al. [9] have proposed a mechanism whereby the lactone ring is opened by means of base-promoted hydrolysis. The substrate bridging allows for optimal positioning of the hydroxide ion for nucleophilic attack on the lactone’s activated carbonyl carbon (through polarization by the zinc ions) [7]. While substrate placement and the general mode of catalysis seem to be agreed upon, certain related elements remain unclear.

Y194 is a key residue whose proposed function we sought to validate. Y194F mutant studies show a loss of 30% activity that has led to multiple proposed explanations [10-13]. Liu (2005) et al. [7] initially described the residue as having either an oxyanion hole function to stabilize the substrate tetrahedral intermediate or as a general acid that protonates the leaving group alkoxide. Next, Kim et al. [7] described Y194 as a general acid as well after conducting a Y194F mutation assay. Liu (2008) et al. [9], upon further investigation, present Y194 as an oxyanion hole provider based on substrate lactone carbonyl oxygen to Y194 OH proton distance as observed during a QM/MM molecular dynamics analysis. In addition, the residue is seen to have a stabilizing effect on the lactone ring and so is identified as a key, binding residue. Last, Liao et al. [8] describe Y194 as an oxyanion hole provider based on quantum mechanics optimization calculations. It is puzzling, however, why an oxyanion hole explanation is favored given the strong, already stabilizing effects of the two Zn (2+) ions that are coordinated to the substrate. Indeed, metalloenzymes such as carboxypeptidase A and some β-lactamases have had substrate hydrolysis mechanisms proposed that do not involve oxyanion hole [14,15].

Proton shuttling is a mechanism by which a proton may be transferred to or from a substrate to a residue on its cognate enzyme to promote a given reaction. Upon completion of the reaction, the proton donor or acceptor is subsequently replenished with a proton from the substrate or from the environment via direct or indirect means. While proximity of a residue to its substrate is one way to qualify it as a candidate shuttle, relative pKa’s are also critical to determining proton disposition. In the case of lactonase, the source of the proton shuttle remains to be fully addressed. In the base-promoted ring-opening mechanism, at least two proton transfers must occur that may or may not directly involve the enzyme. A hydroxide or water must bridge across the two zinc ions. Given the low concentration of hydroxide ions at pH 7, its more likely the water attaches to Zn(2+) first and is subsequently deprotonated given that a metal-bound water molecule’s proton is significantly more acidic than solution bulk water molecule (3-His-Zn(II)-OH_2_ pKa=7.5 vs. H_2_O pKa=15.72) [6]. Furthermore, this water molecule is coordinate by two zin ions, not just one, and is therefore expected to be even more acidic. After the nucleophilic attack occurs by the zinc coordinated hydroxide ion, the tetrahedral intermediate collapses into an anion that must be re-protonated for ease of release from the cationic active site. Both D108 and Y194 are claimed to be proton shuttles in different studies, however, the presence of the active site zinc ions and ligand exchange effects have made it difficult to delineate which, if either, residue performs this function.

**Table 1:** Bioactivities of AiiA toward AHL substrates.

	Activities %	
Substrates	Kim *et al.* 10	Wang *et al.* 5
HSL	ND^a^	5.40
C4AHL	61.4	89.1
C6AHL	100.0	100.0
C8AHL	116.6	93.9
C10AHL	217.1	90.3
C12AHL	31.6	NP^b^

^a^ ND is not detectable

^b^ NP is not performed

Another distinctive feature of the substrate is the acyl tail that is joined to the lactone head via an amide bond. By virtue of the functional groups in the tail, both the amide bond and the alkyl tail may confer activity of the enzyme toward the substrate. Interestingly, contradictory data exist that show both activity dependence and independence on tail length (Table 1) [5,10]. Some of the substrate-enzyme interaction has been attributed to a hydrogen-bond between the backbone NH group of F107 and the substrate amide carbonyl oxygen mediated by a water molecule, however, this interaction does not support a tail-selective activity. Tail-enzyme interactions to date have been described as not strong enough to have an effect on substrate binding given the amount of disorder seen in the alkyl portion in QM/MD simulations. The atomic B-factors derived from the 3DHB crystal structure for the AHL tail are slightly higher (average 29) than the other atoms (average 21) suggesting more relative disorder of the tail. This is not entirely unexpected; however, it is unclear how this translates to functional capability of the ligand’s tail to the enzyme-substrate complex.

## Computational Details

### Protein and substrate coordinates

**Figure 1:**
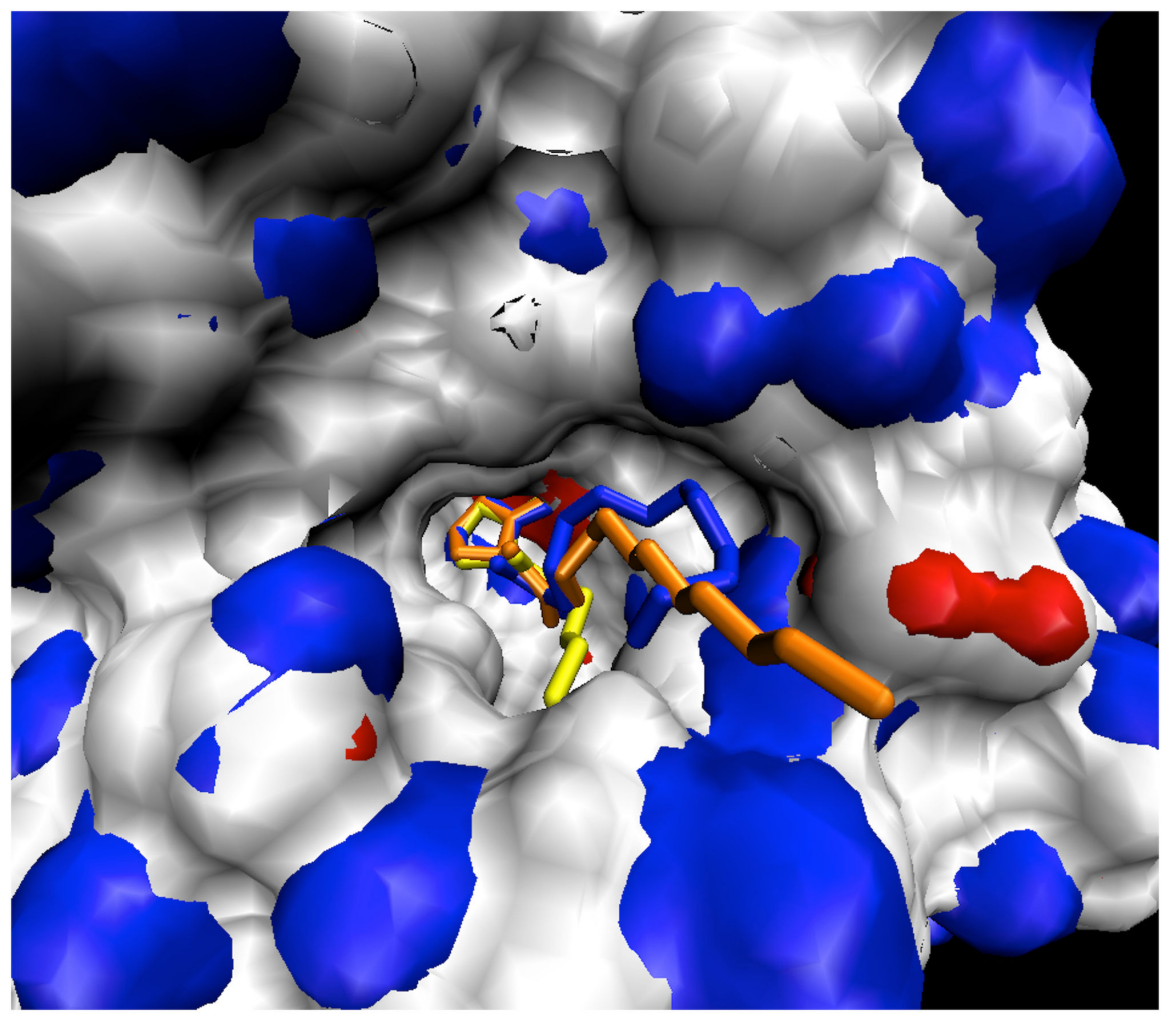
X, Y, and Z conformations of C8-C12AHL represented by the blue, orange, and yellow ligands, respectively.

**Figure 2:**
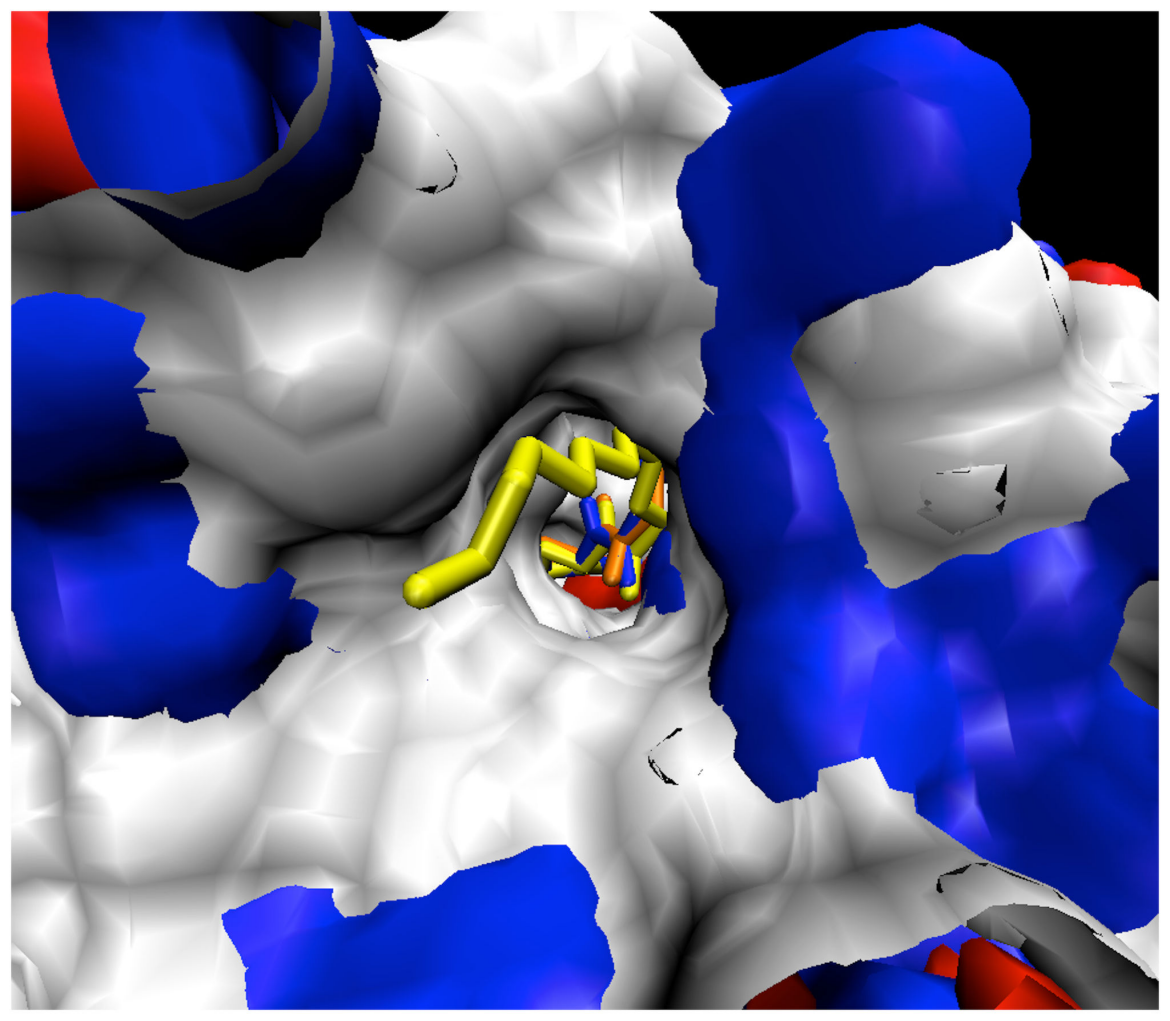
Z conformations of C8-C12AHL tail-end view.

Protein coordinates were obtained from the crystal structure 3DHB [12]. The 3DHB structure was chosen since it possesses a substrate that is well-associated with the active site, thus indicative of an early product state. The substrate coordinates were extracted into SPARTAN [16] where, for the reactant state, the lactone ring was reformed and a constrained minimization was performed to retain the tail coordinates and lactone ring orientation relative to the zinc ions. For the product state, the original ring-opened lactone head (γ-hydroxybutyrate functionality) from the 3DHB structure was retained onto which the alkyl tail was built. In order to observe ligand-enzyme complex behavior pertaining to intramolecular proton transfer, the second intermediate state was constructed. This was achieved by breaking the cyclic ester O-C=O linkage while adding a hydroxide group to the carbonyl carbon. Atomic clashes were removed during the build followed by molecular dynamics energy minimizations. Also, for the reactant state a hydroxyl ion was assigned to occupy the bridging position between the two zinc ions as reasoned from the oxygen density bridging the two metal ions observed in the 2BR6 crystal structure [10]. For C6 and shorter AHLs, the original coordinates from the crystal structure were maintained while for C8 and longer AHLs, three candidate conformations (termed X, Y, and Z) were constructed so as to geometrically sweep out and sample reasonable geography of the pocket in a manner consistent with amide bond orientation and cavity surface geometry. The coordinates from the 3DHB PDB entry were imported into SPARTAN and methyl(ene) groups were added to achieve the desired chain length after which a molecular mechanics minimization was performed on only the methyl(ene) groups to ensure proper bond lengths and angles. These initial minimizations were performed with the AHL ligands in complex with the enzyme and with only the newly added methyl(ene) groups allowed to adjust to their environment. Last, the acyl tail was evaluated to ensure proper C-C bond lengths and angles (Figures 1 and 2). Substrate topologies were generated by the PRODRG2.5 server [17], while substrate partial charges were calculated through ESP fitting with SPARTAN using DFT with the 6-31G* basis set.

### pKa predictions

In an attempt to capture complex conformational effects on active site residue pKa’s, two 4.0 ns molecular dynamics trajectories were run for 3DHB both with and without its *N*-hexanoyl-L-homoserine lactone substrate. The resulting trajectories were then submitted to Jarvis-Patrick (M=7, P=3, cutoff=0.1 nm) cluster analysis as implemented in GROMACS’s g_cluster subprogram where the enzyme with and without ligand produced four and five clusters, respectively. The structures from the trajectory that most closely represented each of the clusters were then submitted to pKa predictions. The protonation states for all ionizable residues in the protein systems were predicted at a pH of 7.0 using the single-site method encoded in the UHBD program [18-20]. The calculations were conducted utilizing a dielectric constant of 80 for water and 20 for the protein. An ionic strength of 150 mM and an ionic radius (for the Stern layer) of 2.0 Å were used. The CHARMM22 polar hydrogen only force field was employed [21]. Hydrogen atoms were added to the molecular systems using the CHARMM program [22] to generate the neutral reference state for the UHBD-based pKa prediction procedure. Both ligand-bound and ligand-free states were analyzed. PROPKA [23-26] was also utilized for pKa prediction as a means to further validate electrostatics based pKa predictions.

### Molecular dynamics simulations and setup

Molecular dynamics simulations were run using GROMACS 3.3 [27] with a GROMOS-96 [28] force field for the protein. The protein was taken from PDB entry 3DHB from which all crystal waters were retained. The 3DHB structure was specifically chosen as it more clearly represents an early enzyme-product state. All protein substrate complexes were centered in a water box with simple point charge (SPC) [29] waters such that none were within 3.5 Å of any existing atom and water molecules extended 10 Å beyond the furthest protein atom in each Cartesian direction. The total system charge was neutralized using the appropriate number of Na+ or Cl- counter-ions. An energy minimization was first performed to remove interatomic clashes using 500 steps of the steepest descent algorithm. Next, position restrained molecular dynamics was run for 100 ps allowing both the substrate and waters to relax about the restrained protein. A set of 1.0 ns production molecular dynamics simulations were carried out for each of the ligand-protein complexes. An additional 4.0 ns production run was performed to generate more statistical data. All simulations were run at a temperature of 300K in the NPT ensemble with a 2 fs timestep employing periodic boundary conditions and the LINCS algorithm [30] to constrain bonds to hydrogen atoms. Both Berendsen pressure and temperature baths [31] were employed with relaxation coefficients of 0.1 and 1.0 ps, respectively. Lastly, long-range electrostatic interactions were managed using particle-mesh Ewald methodology beyond a cutoff of 9.0 angstroms.

### Distance Analyses

**Figure 3:**
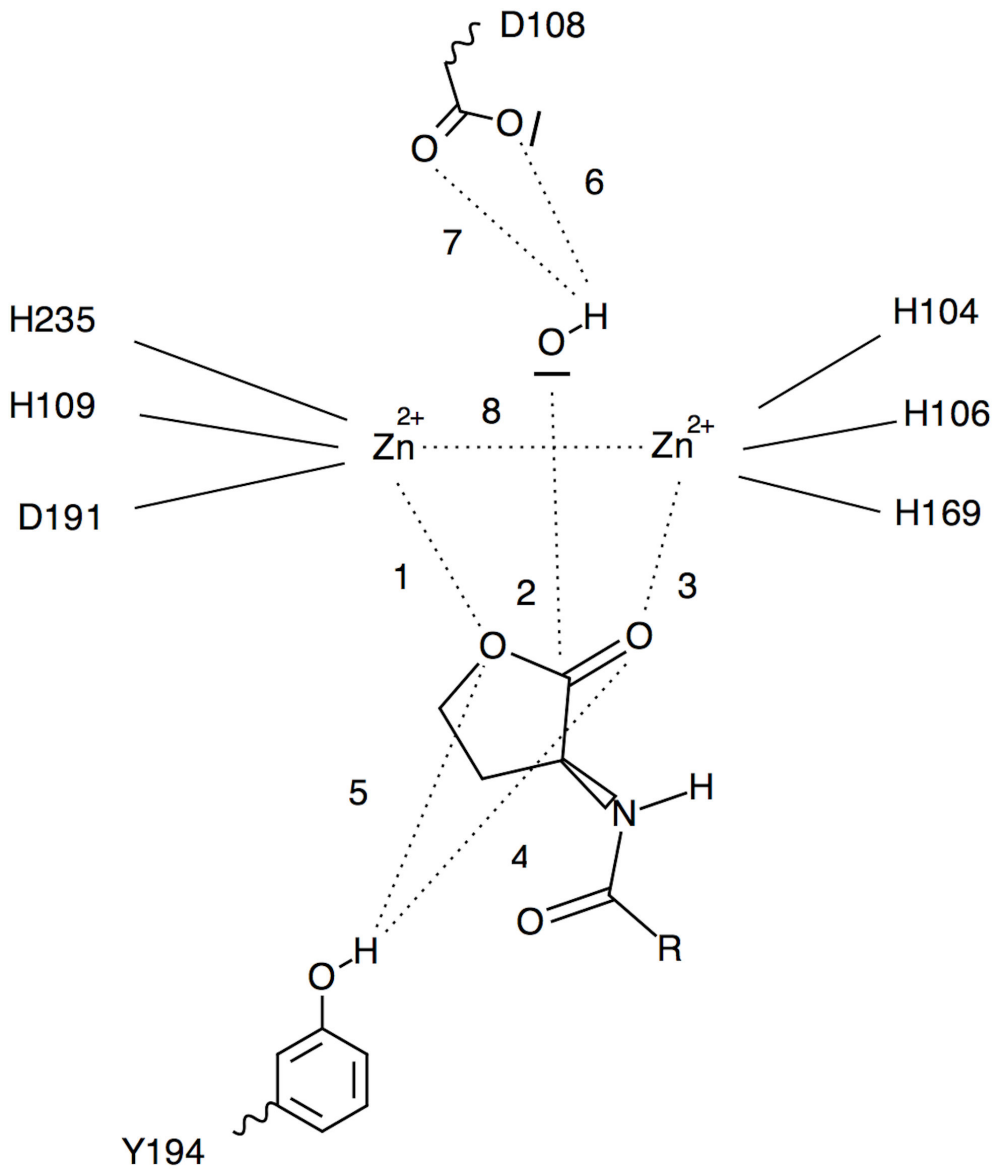
Reactant state distance map.

**Figure 4:**
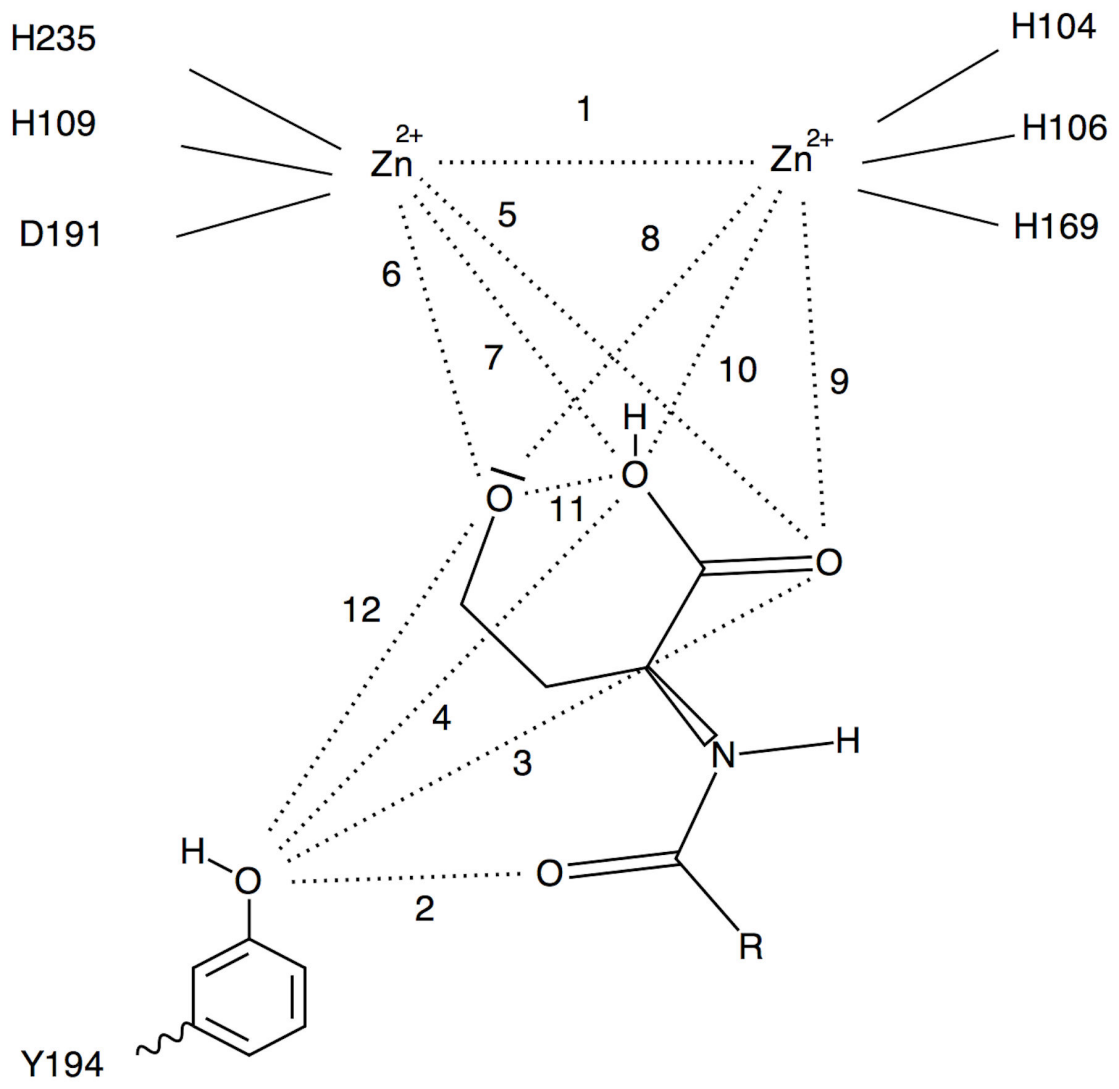
Intermediate state distance map.

**Figure 5:**
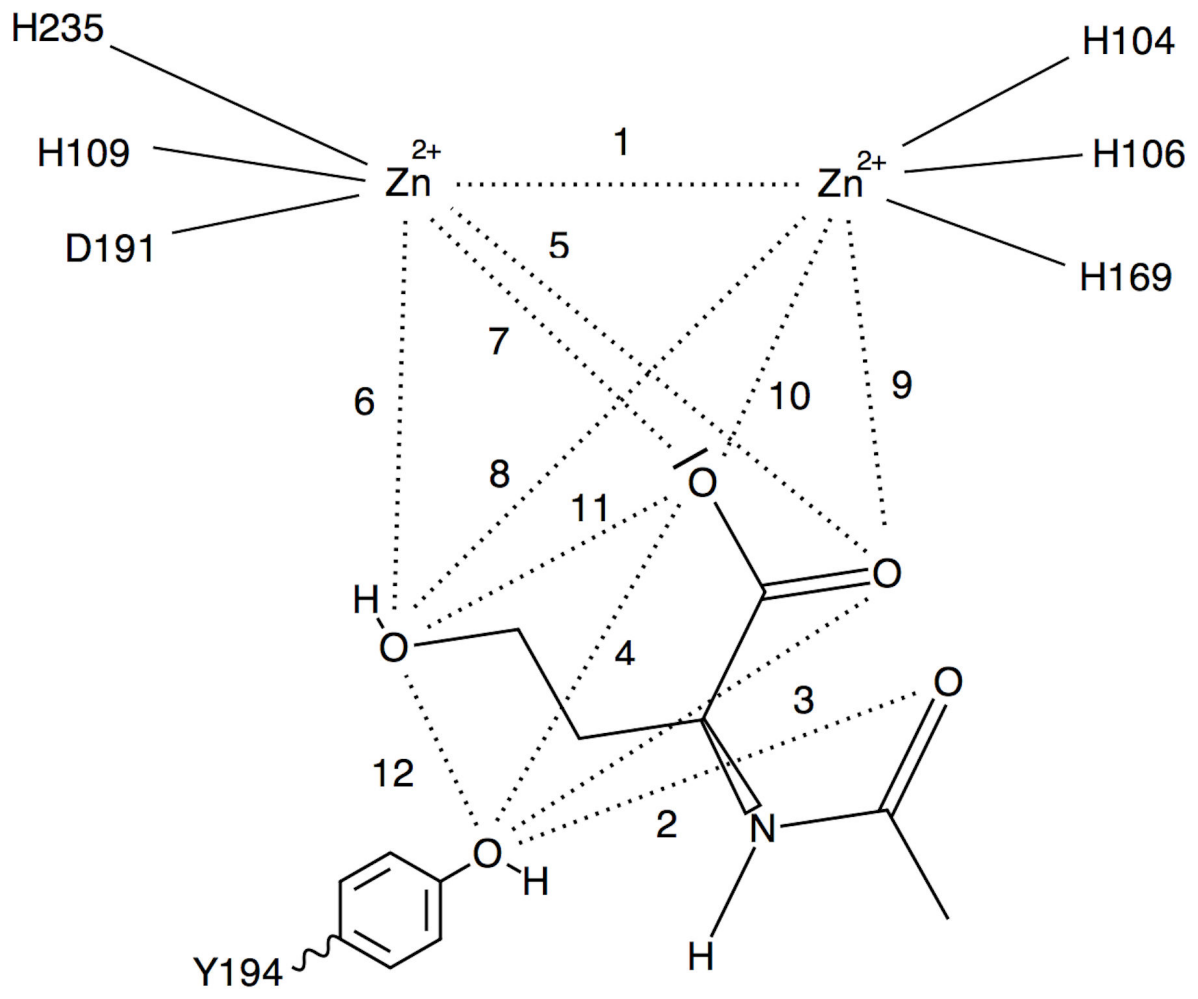
Product state distance map.

Interatomic distances across MD trajectories were calculated to ascertain catalytic competence and other residue contributions using GROMACS’ g_dist program [27]. Reactant, intermediate, and product state distances were all analyzed (Figures 3, 4, and 5, respectively). Specifically, distances 1, 2, and 3 of the reactant state were observed to monitor the lactone ring bridging the zinc atoms. Distances 4 and 5 of the reactant state were observed to investigate the contribution of Y194 to ligand stabilization and/or early tetrahedral intermediate stabilization. Distances 6 and 7 of the reactant state were investigated to study how D108 might play a role in hydroxide ion stabilization. Intermediate and product state residue contributions were also investigated in a likewise manner and focused on residue-ligand interactions that point to early or late effects of transition state stabilization.

### Interaction Energies

For van der Waals interaction energy analysis of protein-ligand interactions in the enzyme complex, the Q program [32] was utilized. For these cases, end-state conformations were obtained from the GROMACS production run trajectories as starting states for the Q trajectories. For these calculations the CHARMM22 force field as implemented in Q was used. Q was specifically written to do linear interaction energy (LIE) calculations and was utilized in this case to facilitate long-range electrostatic interaction energy accounting. The CHARMM22 force field, as implemented in Q, was most amenable to our needs with regards to ligand parameterization. For the purposes of trend analysis, we expect no significant discrepancies between the GROMOS-96 and CHARMM22 force fields. The frames were captured every 75 fs. A 15 Å water solvated boundary sphere was implemented centered on zinc 252 (per 3DHB) to include the ligand and active site. A heat-up procedure was run for 160 ps from 50K to 300K with restrained ligand and protein followed by a 1.0 ns production run.

Single-point energy calculations on truncated substrate and active site residues were conducted with GAUSSIAN 03 [33] using DFT with a B3LYP functional. The 6-31G(d, p) basis set was employed. The active site components for these calculations consisted of both zinc ions, Y194, D108, D191, H104, H106, H109, H169, and H235. All residues were methylated and frozen at the alpha carbons. The substrates were terminated with a methyl group after the amide group carbonyl carbon. The single point energy analyses were conducted on substrate poses that were observed to be well-represented (sustained for at least 1 ns) over all the reactant, intermediate, and product state trajectories. All data and trajectories are available upon request.

**Table 2:** Ligand-protein average distances (in Angstroms) of quorum signaling ligands in complex with AiiA measured from molecular dynamics simulations. Distances are defined in Figure 3. Tail length and X, Y, and Z conformations are denoted for each applicable ligand.

**Ligands**	**Distance 1**	**Distance 2**	**Distance 3**	**Distance 4**	**Distance 5**	**Distance 6**	**Distance 7**	**Distance 8**
**HSL**	4.1±0.9	3.7±0.2	2.1±0.1	4.1±0.5	3.3±0.9	3.7±0.1	3.8±0.2	3.5±0.1
**C4AHL**	4.1±0.3	3.6±0.3	2.1±0.1	6.3±0.7	5.7±0.7	3.6±0.1	3.8±0.1	3.6±0.1
**C6AHL**	4.2±0.3	3.1±0.1	2.1±0.1	3.2±0.3	2.4±0.4	3.0±0.3	2.2±0.3	3.5±0.1
**C8X_AHL**	4.0±0.3	3.2±0.1	2.1±0.1	3.8±0.3	4.3±0.4	3.4±0.8	2.7±0.5	3.6±0.1
**C8Y_AHL**	3.8±0.3	2.9±0.1	3.8±0.2	3.8±0.3	4.3±0.4	3.4±0.8	2.7±0.5	3.5±0.1
**C8Z_AHL**	3.9±0.3	3.1±0.1	2.1±0.1	3.2±0.5	3.0±0.4	2.7±0.4	2.8±0.4	3.5±0.1
**C10X_AHL**	3.7±0.4	3.6±0.3	2.1±0.1	4.8±0.8	3.7±1.1	3.6±0.1	3.8±0.1	3.5±0.1
**C10Y_AHL**	4.0±0.2	3.8±0.2	4.0±0.2	3.8±0.6	4.9±1.0	4.1±0.2	2.7±0.2	3.5±0.1
**C10Z_AHL**	3.8±0.3	3.1±0.2	2.1±0.1	5.9±1.2	5.3±1.4	2.5±0.5	2.4±0.6	3.6±0.1
**C12X_AHL**	4.2±0.6	3.7±0.3	2.0±0.1	4.7±0.5	3.9±0.9	3.6±0.1	3.8±0.1	3.6±0.1
**C12Y_AHL**	4.1±0.1	4.1±0.1	4.1±0.2	3.5±0.4	4.1±0.5	3.9±0.2	2.4±0.2	3.5±0.1
**C12Z_AHL**	3.6±0.3	3.1±0.1	2.1±0.1	5.4±0.3	5.6±0.4	2.8±0.5	2.8±0.5	3.4±0.1

## Results and Discussion

### Active site configuration (distance analysis)

To achieve the proper bridging position for the reactant state, the substrate ring oxygens should orient across each zinc ion in the active site at a consistently close proximity. The resulting geometry would in turn provide for a near-attack positioning of the hydroxide ion oxygen to the lactone ring carbonyl carbon thus promoting an optimal nucleophilic attack trajectory. Table 2 compares homoserine lactone (HSL) to the other reactant AHLs to compare ligands with and without tails to investigate the function of the tail in inducing bioactivity of the enzyme toward the substrate. Across the HSL, C4AHL, C6AHL, and the X and Z conformations of the C8-C12AHL species, distance 3 (Table 2) is seen as the shortest and most stable. The stability and proximity derives from the fact that the carbonyl oxygen carries a more negative partial charge and least steric hindrance of the two lactone ring oxygen atoms. The Y conformation of the C8-C12AHL species exhibits a doubling of distance 3. Close inspection of the equilibration MD trajectories revealed that, in each case, a water molecule comes into close enough contact with the zinc atom as to displace the carbonyl oxygen to the alternate zinc ion. This observation is not surprising as the SPC water molecule oxygen is more negatively charged than the carbonyl oxygen. Distance 1 shows the lactone ring ether oxygen atom residing at 1.5 to 2 times the length of distance 3. This can be explained by the fact that the lactone ring ether oxygen carries less partial negative charge and is more sterically hindered than the carbonyl oxygen. The overall span of distance 1 across all ligands is consistent. The C8-C12AHL Y conformations for distance 1 exhibit less difference across each ligand as the ether oxygen and displaced carbonyl oxygens both ligate the same zinc ion. Distance 2 exhibits consistent and stable values spanning 3.1-3.7 angstroms across HSL, C4AHL, C6AHL, and the X and Z conformations of the C8-C12AHL ligands. These distances appear close enough to promote nucleophilic attack of the hydroxide ion on the carbonyl carbon of the lactone ring. The Y conformations of C8-C12AHL ligands reveal a notably longer distance 2. This longer distance, in addition to the loss of the bridging position of the lactone ring oxygens across the two zinc ions, suggests the loss of catalytic competence for the C8-C12AHL Y conformations. We reason the observed Y conformation shift therefore reveals a probability that water molecules might be retained or enter the active site so as to shift the ligation pattern of the substrate that is critical to catalytic turnover

**Table 3:** Distance analyses of intermediate state ligands in Angstroms. **Distances are defined in Figure 4**.

**IS-Ligands**	**Distance 1**	**Distance 2**	**Distance 3**	**Distance 4**	**Distance 5**	**Distance 6**	**Distance 7**	**Distance 8**	**Distance 9**	**Distance 10**	**Distance 11**	**Distance 12**
**C4AHL**	4.8±0.8	6.9±0.6	5.5±1.0	5.7±0.8	6.7±0.6	1.9±0.0	7.1±0.3	4.7±0.8	6.5±1.8	6.6±0.9	5.5±0.2	3.5±1.0
**C6AHL**	4.2±0.2	5.9±0.3	3.1±0.2	4.4±0.2	4.3±0.2	1.9±0.0	3.3±0.2	4.1±0.1	2.0±0.1	2.2±0.1	3.1±0.1	2.8±0.2
**C8X_AHL**	4.5±0.5	6.6±0.4	3.1±0.3	4.6±0.4	4.8±0.3	1.9±0.1	3.9±0.6	3.7±1.0	2.0±0.1	2.6±0.7	3.2±0.4	3.4±0.6
**C8Y_AHL**	4.8±0.2	6.6±0.5	3.2±0.4	4.6±0.5	4.8±0.2	1.9±0.0	3.7±0.2	4.3±0.2	2.0±0.1	2.2±0.1	3.1±0.2	3.6±0.7
**C8Z_AHL**	3.6±0.1	6.0±0.4	2.7±0.2	4.4±0.6	4.6±0.3	2.0±0.1	5.8±0.7	2.0±0.3	2.1±0.1	4.0±0.3	4.3±0.4	3.0±0.3
**C10X_AHL**	5.4±0.6	6.0±0.7	2.9±0.3	4.0±0.9	5.2±0.6	1.9±0.0	3.8±0.3	4.5±0.3	2.0±0.1	2.6±0.6	2.9±0.4	4.2±0.7
**C10Y_AHL**	4.6±0.3	6.5±0.9	3.3±0.3	4.8±0.5	4.6±0.3	1.9±0.0	3.5±0.2	4.4±0.3	2.0±0.1	2.2±0.2	3.1±0.2	4.1±1.0
**C10Z_AHL**	5.3±0.1	5.7±0.5	4.3±0.4	4.1±0.4	2.1±0.1	1.9±0.0	4.2±0.1	5.9±0.2	3.7±0.1	2.2±0.1	4.4±0.2	4.5±0.4
**C12X_AHL**	4.8±0.2	6.3±0.3	3.1±0.2	4.2±0.2	4.9±0.2	1.9±0.0	3.2±0.2	4.3±0.2	2.0±0.1	2.2±0.1	3.1±0.2	2.9±0.3
**C12Y_AHL**	4.8±0.2	5.7±1.0	3.2±0.3	4.5±0.4	4.8±0.3	1.9±0.0	3.7±0.2	4.3±0.2	2.0±0.1	2.2±0.2	3.2±0.2	3.5±0.8
**C12Z_AHL**	4.3±0.4	5.6±0.7	5.2±0.3	6.9±0.6	3.7±0.4	1.9±0.0	3.8±0.5	3.8±0.5	2.1±0.3	4.1±0.4	4.2±0.2	4.7±0.4

Distances 4 and 5 were analyzed through the MD trajectories to see how Y194 might contribute to stabilizing the lactone rings of the AHL ligands through hydrogen-bonding to the carbonyl oxygens or ether oxygens, respectively. Distance 4 exhibited high variability across ligands with proton to oxygen distances well outside those of hydrogen-bonding range. Distance 5 also exhibited high variability across ligands with proton to oxygen distances well outside those of hydrogen-bonding range. In one of the trajectories, a similar analysis performed on the Y194 oxygen to amide carbonyl oxygens revealed a trajectory with a tight, persistent, distance of 2.4 ± 0.1 Å and a corresponding O-H-O angle of 158° ± 10°. The corresponding O-H-O angle distribution was consistent with that of a hydrogen-bond throughout the trajectory. This potential hydrogen-bond was not seen in the initiating structure or in the energy minimization or soaking steps, but was formed early in the production simulation.

**Table 4:** Distance analysis of product state ligands in Angstroms. Distances are defined in Figure 5.

**PS-Ligands**	**Distance 1**	**Distance 2**	**Distance 3**	**Distance 4**	**Distance 5**	**Distance 6**	**Distance 7**	**Distance 8**	**Distance 9**	**Distance 10**	**Distance 11**	**Distance 12**
**C4AHL**	4.7±0.4	4.5±0.6	3.8±0.6	2.9±0.2	2.3±0.4	2.1±0.1	4.3±0.3	5.2±0.5	2.8±0.4	2.1±0.1	4.2±0.6	5.0±0.7
**C6AHL**	4.9±0.2	6.7±0.8	4.8±0.8	3.6±0.5	3.7±0.2	2.0±0.1	5.1±0.2	4.4±0.2	2.2±0.1	2.1±0.1	5.7±0.6	3.6±1.1
**C8X_AHL**	3.8±0.1	5.9±0.7	5.5±0.5	3.8±0.4	2.3±0.1	2.0±0.1	3.9±0.1	4.6±0.1	2.4±0.1	2.1±0.1	6.9±0.4	5.3±0.7
**C8Y_AHL**	4.9±0.3	5.0±1.0	4.6±0.5	4.6±1.1	3.4±0.3	4.5±0.5	2.1±0.1	6.7±0.5	2.2±0.1	3.9±0.3	7.0±0.5	6.7±1.0
**C8Z_AHL**	5.1±0.1	6.6±0.6	5.0±0.5	5.2±0.6	3.9±0.1	4.5±0.3	2.1±0.1	7.0±0.4	2.1±0.1	3.7±0.1	7.3±0.3	7.0±1.1
**C10X_AHL**	3.8±0.1	6.5±0.7	5.9±0.6	4.1±0.5	2.3±0.1	2.0±0.1	3.8±0.1	4.6±0.1	2.3±0.1	2.1±0.1	7.1±0.4	5.4±0.7
**C10Y_AHL**	5.2±0.1	5.6±0.3	4.4±0.3	4.4±0.4	3.9±0.1	4.5±0.3	2.1±0.1	7.3±0.3	2.1±0.1	3.7±0.1	7.0±0.5	6.3±0.5
**C10Z_AHL**	5.0±0.2	6.6±0.9	4.8±0.5	5.4±1.0	3.5±0.2	3.2±1.5	2.1±0.1	6.0±0.7	2.2±0.1	4.1±0.2	7.1±0.4	5.8±1.1
**C12X_AHL**	3.8±0.2	7.1±0.7	5.1±0.7	3.7±0.5	2.3±0.2	2.0±0.1	4.0±0.2	4.6±0.1	2.3±0.1	2.1±0.1	7.1±0.4	4.6±0.9
**C12Y_AHL**	4.3±0.3	7.1±0.8	5.3±1.2	5.4±1.6	3.0±0.4	5.9±0.8	2.1±0.1	7.8±0.7	2.5±0.6	2.5±0.6	7.0±0.4	8.6±1.7
**C12Z_AHL**	4.9±0.2	7.6±0.7	5.8±0.6	5.6±0.6	3.7±0.2	4.8±0.5	2.1±0.1	6.8±0.3	2.2±0.1	3.6±0.1	7.0±0.4	7.3±0.8

Distances 6 and 7 were examined to determine how D108 might influence the stability of the hydroxide anion. Distance 6 exhibited a fairly wide variance and longer distance when compared to distance 7. Distance 7 shows the hydroxide proton to aspartate oxygen distances that come to within a hydrogen-bonding distance across many of the ligands and conformations. The distances appear to become more consistent through the C6-C12AHL species. Furthermore, these distances combined with the hydroxide bridging position across the zinc atoms makes for a stable network by which to ensure an incoming substrate is presented in the best position for nucleophilic attack, provided that the substrate can achieve its own bridging orientation and appropriate binding affinity.

### Intermediate and product state configurations

In the intermediate and product states (Tables 3 and 4, respectively), the zinc-to-zinc distance 1 (Figures 4 and 5) is significantly longer and more variable than seen in the reactant state. One possible reason for this distance increase is that upon ring opening, the resulting alkoxide and carboxyl oxygens that bridge the two zinc ions are now four carbons apart and able to move more freely to allow for different modes of metal coordination. The broadening of the zinc-to-zinc distance also provides for more water access to the zinc ions. Whereas in the reactant state, the C8-C12AHL Y conformations only exhibit a water molecule ligated to a zinc, in both the intermediate and product states about 50% of all complexes exhibit one or more waters accessing the carboxyl(ate) bound zinc ion suggesting at least part of a means for product release.

Another observation is the alkoxide oxygen – (carboxyl oxygen length shown in distance 11) of both the intermediate and product states. The intermediate state distances are significantly shorter than those seen in the product state that approaches twice that of the latter. This is significant as it suggests that the functional group ends are close enough to allow for an intramolecular proton transfer. Further investigation into this phenomenon reveals that the Y194 oxygen to intermediate state alkoxide oxygen distance 12 is close enough to create a stabilizing effect. The same stabilizing effect is not seen in the product state. When put together, these observations suggest that Y194 stabilizes the second transition state in addition to or instead of the first transition state.

### pKa prediction results (Y194, D108)

pKa predictions performed on both the enzyme-substrate complex and without hexanoyl-*L*-homoserine lactone did not reveal any significant perturbations to the pKa’s of the Y194 or D108 residues. In the cases where the ligand was included, the molecule was treated as a non-ionizable residue. As the Y194 and D108 residues are considered candidate proton shuttles for the zinc-bridging water, it is noteworthy that the pKa study revealed no shift of their pKa’s toward that necessary to deprotonate a metal-bound water (that being around 7.5). Y194 pKa’s averaged 11.6±0.1 across ligated and unligated protein states while D108 and D191 titrated below their reference pKa of 4.4. That D108 and D191 remain in their anionic state is not surprising given their proximity to the zinc di-cations, thus validating their function as metal ligands. H104, H169, H104, H235, and H109 were also found to be in their neutral state, consistent with their role in metal ion coordination. Results were also consistent between two computational approaches (UHBD [18-20] and PROPKA [23-26]).

**Figure 6:**
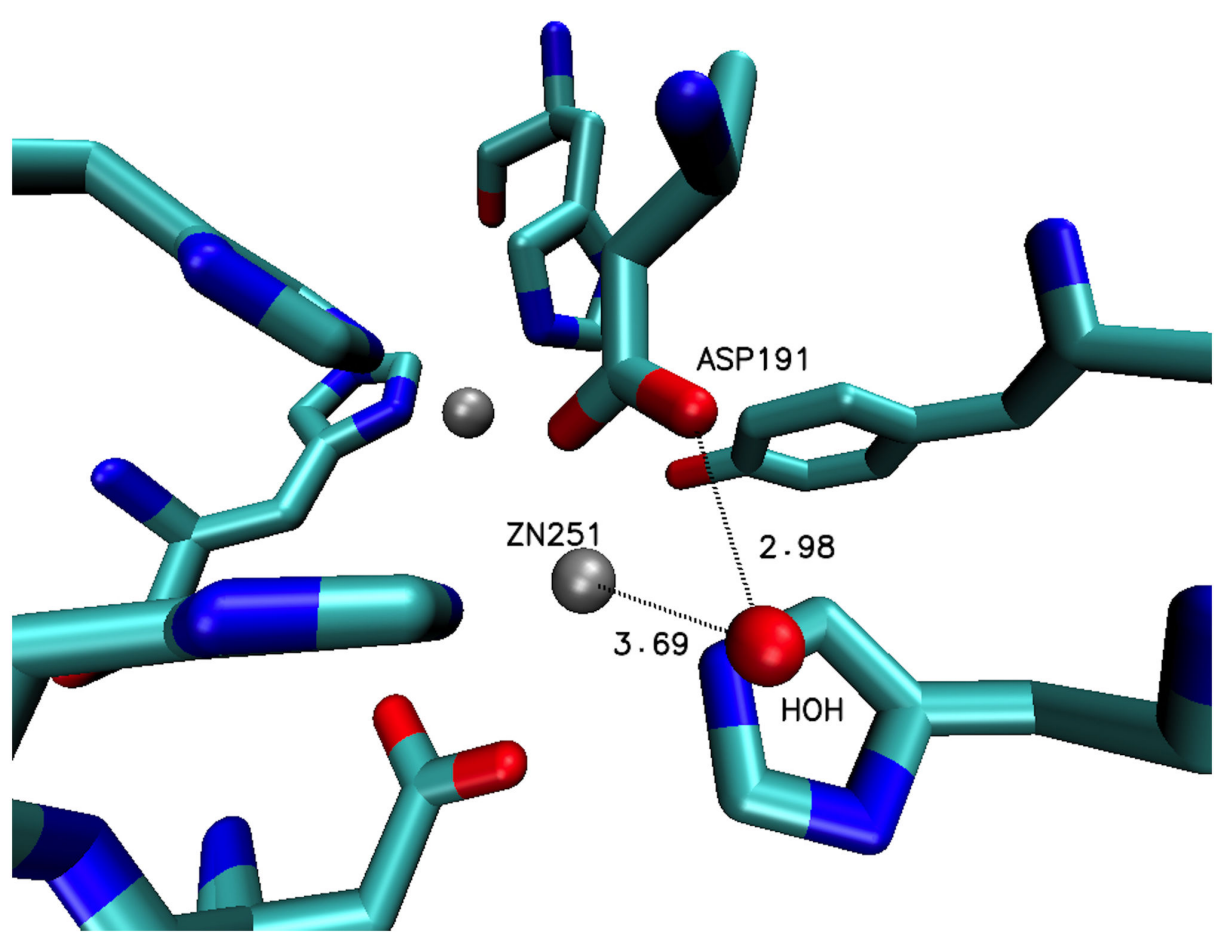
3DHB crystal structure with water molecule in proximity to Zn251 and D191.

Further study of the 3DHB crystal structure revealed an oxygen atom located 3.7Å from ZN 251 and 3.0 Å from the carboxylate oxygen of D191 (Figure 6). To investigate the effect of a water molecule at this location, pKa analyses were conducted with PROPKA and H++ 3.0 [34-36]. As in the water-free pKa analyses, both methodologies revealed both D108 and D191 titrating well below their reference pKa’s. Y194 as well titrated very closely to the water-free states. Lastly, the C6AHL product state (essentially 3DHB derived) MD trajectory showed a water molecule in similar proximity to the metal ions, D108, and D191 at the beginning of the run only to migrate to about 10 Å away at the end thus demonstrating low occupancy.

### Interaction energy analysis versus bioassays

An interaction energy study was performed across all reactant, intermediate, and product states to gain a fuller understanding of how they might better inform us of the discrepancy seen between the two sets of bioassay data. As our primary interest was the potential for tail-mediated bioactivity, a series of analyses was conducted on the van der Waals interaction energies across all ligand-enzyme complexes using the Q program (Table 5). As HSL has no tail and as it has not been identified as bioactive with regards to quorum sensing, it was excluded from this analysis. It was observed that the reactant state van der Waals energies were more consistent across the reactant states with respect to tail length than across the intermediate and product states that showed more variability. Similar trending was seen with regards to electrostatic energies across all reactant states where values were much closer to one another. The intermediate and product state electrostatic energies were higher relative to the reactant states and more variable. We reason this trending is due to the addition of the hydroxyl group and increase in degrees of freedom of the substrate due to ring-opening.

**Table 5:** van der Waals interaction energies in kcal/mol.

**Ligand**	**Reactant State**	**Intermediate State**	**Product State**
**C4AHL**	-13.6±0.3	-4.5±7.5	3.9±1.4
**C6AHL**	-12.9±0.9	-5.1±0.5	-0.6±0.3
**C8X_AHL**	-13.2±0.7	-0.2±1.8	-3.7±0.8
**C8Y_AHL**	-12.9±0.1	2.3±0.8	-2.7±3.2
**C8Z_AHL**	-16.3±0.4	0.4±0.6	-4.5±1.5
**C10X_AHL**	-14.3±0.8	-4.6±1.1	-0.3±1.2
**C10Y_AHL**	-14.5±0.4	-3.5±0.4	-5.7±1.8
**C10Z_AHL**	-16.8±0.3	-0.7±0.7	-4.2±0.4
**C12X_AHL**	-21.6±0.4	-4.4±0.4	-4.2±1.1
**C12Y_AHL**	-12.5±0.9	-0.4±2.2	-2.8±1.0
**C12Z_AHL**	-17.0±0.4	2.4±0.1	-3.3±1.2

The changes in van der Waals interaction energy of the reactant states per mole of methylene addition were calculated to be 0.43 kcal/mol, agreeing well with 0.38 kcal/mol per methylene addition seen in *n*-alkyl alcohols in water/*n*-octanol partitioning experiments exploring substituent effects in enzyme-substrate complexes [37]. Lastly, the per mole methylene energy addition agrees well with previous calculations across the same species where the energy delta was calculated to be 0.24 kcal per mol methylene increment based on *k*_cat/_/K_m_. values [38]. The van der Waals interaction energies of the intermediate and product states, while noisier, offer no information to refute trending observations made on the reactant states suggesting no dependence of enzyme-substrate activity toward substrate tail length.

### Dizinc ion ligation

Another point of interest was to consider what other interactions might be occurring between the substrate head (lactone or n-hexanoyl homoserine) and the active site. To that effect, all MD trajectories across all states we examined with a focus on substrate head orientation relative to the active site. Snapshots of poses that were observed to be distinct in space and sustained in time (greater than 1.0 ns) were extracted. The 20 poses across (5-7 poses per state) all states were subject to B3LYP/6-31G* single point quantum mechanical energy calculations to provide for a rough energy sampling and trending across all states (see Figure S1, S2, S3 for representative figures). These calculations were undertaken understanding that prior QM optimizations had been performed on the active site only with the residue side chains frozen at the alpha carbons as per the crystal structure. We reasoned that physically, the complex is subject to thermal motion and, in this case (as it is a metalloenzyme), subject to ligand exchange). The aim was to roughly sample this space. Each of our poses were subject to the same measurements as the MD trajectories to see if there were any pose-induced energy sensitivity trends that could be rationalized. The most discernible trend was a general increase in zinc to zinc distances as the structures became more energetically favorable across all states. An investigation of interatomic distances revealed that the zinc to zinc ion distances were on average 3.5±0.1 Å for the reactant states while the same distance for the product and intermediate states was 4.7±0.7 Å. That the intermediate and product state poses were found more stable under these zinc to zinc distances is reasonable given the additional degrees of freedom of the substrate relative to the reactant states allowing for better ligation of the zinc ions.

Last, we compared our data to experimental EXAFS data derived from L1, a class 3 metallo-β-lactamase (MβL) also containing dizinc 2+ ions subject to a similar ligation field to AiiA [39]. In this study, rapid freeze quenching of the MβL–substrate complex was utilized to examine the metal-to-metal distance of the dizinc ions. Resting, intermediate (quenched 10 ms after reaction initiation), and product states were obtained for EXAFS analyses. The metal-to-metal distances for the resting, intermediate, and product states were 3.42, 3.72, and 3.62 Å, respectively. By comparison, our data were 3.52, 4.65, and 4.58 Å for the same states across all of our ligands (C4AHL-C12AHL). Our reactant state data were very close to the experimental data while the intermediate and product states were longer, however, the same relatively small difference between the intermediate and product states trended well. We reason that the longer distance is due to the ring-opening of a five versus a four-membered ring where the additional methylene group necessarily introduces two additional C-C bonds, an angle, and a torsion thus allowing for the metal ions to move further from each other. We reason that these observed similarities and rationalized differences speak well to the fidelity of our simulations. In addition, we see these observations as critical to better understanding the effect of ligand exchange that prior quantum mechanical optimizations have not more explicitly addressed as they can only optimize to singular solutions and cannot account for the statistical variation expected even at an energy minimum.

## Conclusion

The AiiA lactonase enzyme produced by *B. thuringiensis* exhibits bioactivity toward the quorum signaling molecules of Gram-negative bacteria in what appears to be an evolved mechanism by which one organism can compete with another. The quorum signal degradation occurs through action of the AiiA enzyme in what is seen as an activity that may be tail-length dependent. The bioassay data however, are inconsistent and the key interactions are not well understood. To gain more insight into the enzyme-substrate interactions, we performed multiple MD simulations across a family of Gram-negative quorum signaling molecules, acyl homoserine lactones. These simulations were careful to include the AHLs in their reactant, intermediate, and product complexed states.

We observed consistent catalytic competence with regard to geometric distances across the lactone ligands with the Y-conformation of the C8-C12AHL ligands standing out as one that demonstrates how important water exclusion is to enzyme function. Water exclusion is a critical and common theme in enzymology and so it is of no surprise that some conformations may be favored over others to this effect. The tail appears to serve as a “plug” barring water access to the active site. It was further observed that half of the intermediate and product states retained water at the zinc ions. This was facilitated by the ring-opened ligand providing for the enzyme to “breath” thus allowing the zincs to move further apart to allow for water ligation. Next, we produced van der Waals interaction energy data that correlate well with bioassays suggesting that the tail only crudely orients the lactone head in the active site. Thus, the full range of van der Waals ΔG’ s for lactone ligands is consistent with the bioassay data observed by Kim et al. [10]

**Figure 7:**
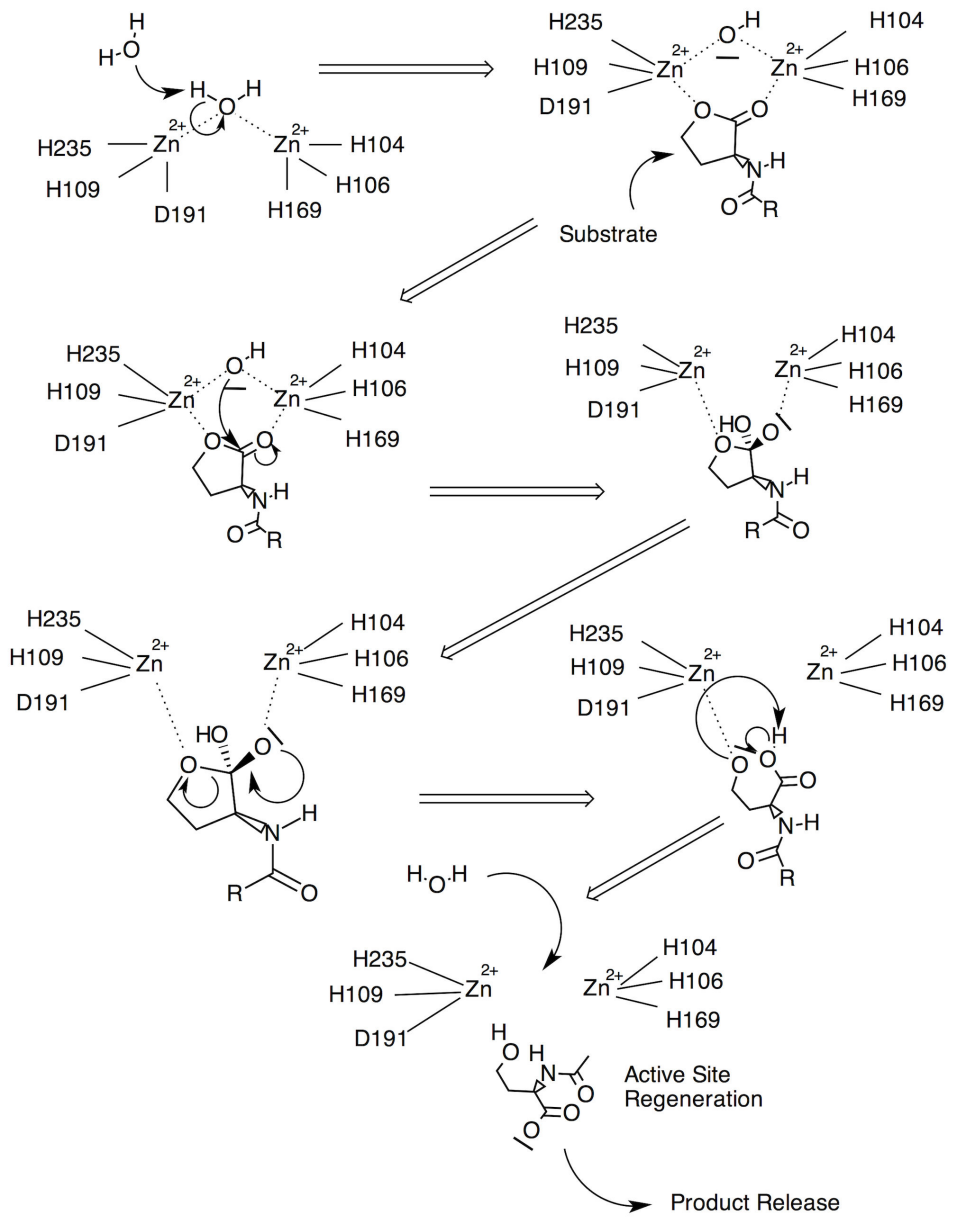
Proposed mechanism.

Residues D108 and Y194 were investigated for their influence on binding energetics and substrate stability. Also, both D108 and Y194 are referred to as having the potential to serve as proton shuttles for the zinc bound water and protonation of the ring opened alkoxide and so were examined for that effect. The distance analysis of Y194 showed no early potential in forming an oxyanion hole. This outcome may be due to the simulations with reactants that have less developed negative charge on the lactone carbonyl oxygen (q = -0.46) than that of the anionic transition state (closer to -1.0). That the reaction is not totally quenched by the Y194F mutation suggests that Y194 serves as a stabilizing influence rather than as a true oxyanion hole. This is strengthened by observations of the influence of Y194 on the intermediate state carbonyl oxygen but not on the reactant or product state. That Y194 influences the intermediate state suggests late involvement in the first transition state or conversely, early involvement in the second. D108 is observed to be close enough to the hydroxide ion to perhaps provide some stabilizing influence through hydrogen-bonding. Lastly, pKa analysis showed no evidence of either D108 or Y194 having shifted pKa’s in the range necessary to serve as effective proton shuttles for the zinc-bound water and ring-opened alkoxide. However, this does not preclude water itself from serving as the (de)protonating medium. The pKa of bulk water is 15.7 which is more than basic enough to deprotonate a zinc bound water (pKa 7.5), whereas the resulting hydronium ion is definitely acidic enough (pKa -1.7) [40] to protonate adjacent waters. Moreover, studies of similar dimetalloproteins and dimetallic enzyme analogs suggest that the pKa of a water molecule bridging zinc ions is lowered to no more than about 5.8 - still two orders of magnitude higher than D108’s acid dissociation constant [41,42]. Inspection of the binding pocket reveals a structure that is stable and well exposed to its environment thus providing an avenue for proton transport though a water molecule network that exists prior to substrate binding. Upon substrate binding, the zinc-bridging hydroxide anion is effectively protected from re-protonation by the plug-like function of the acyl tails whereupon the ring-opening reaction can proceed. Subsequent protonation of the product’s alkoxide group can then be accomplished by the intramolecular proton transfer from the intermediate state’s carboxylic acid group where the resulting carboxylate group better accommodates the still-neighboring zinc ion. The proposed mechanism supporting these aforementioned issues is rendered in Figure 7.

## Supporting Information

Figure S1 Front side of active site, no substrate. (TIFF)

Figure S2 Back side of active site, no substrate. (TIFF)

Figure S3 Ring-opened product. (TIFF)
