# Supplementary figures and images for: New Insights into the Binding and Catalytic Mechanisms of Bacillus thuringiensis Lactonase: Insights into B. thuringiensis AiiA Mechanism
Source: PLoS One. 2013 Sep 18;8(9):e75395. doi: 10.1371/journal.pone.0075395 (PMC3776789; doi:10.1371/journal.pone.0075395)

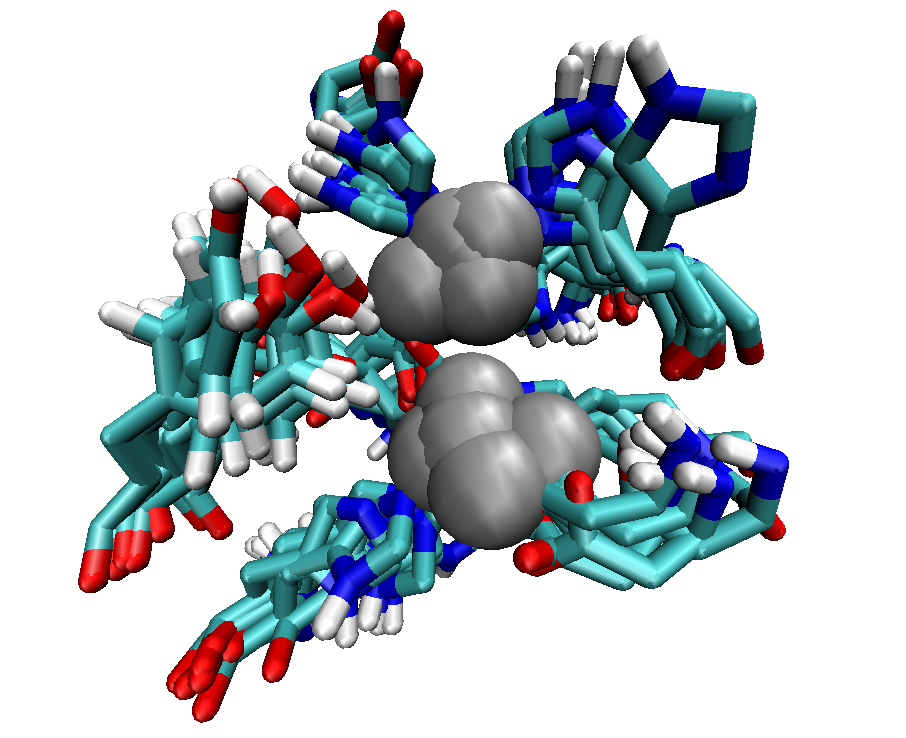

Supplement: Figure S1 — (TIFF) [file pone.0075395.s001.tiff]

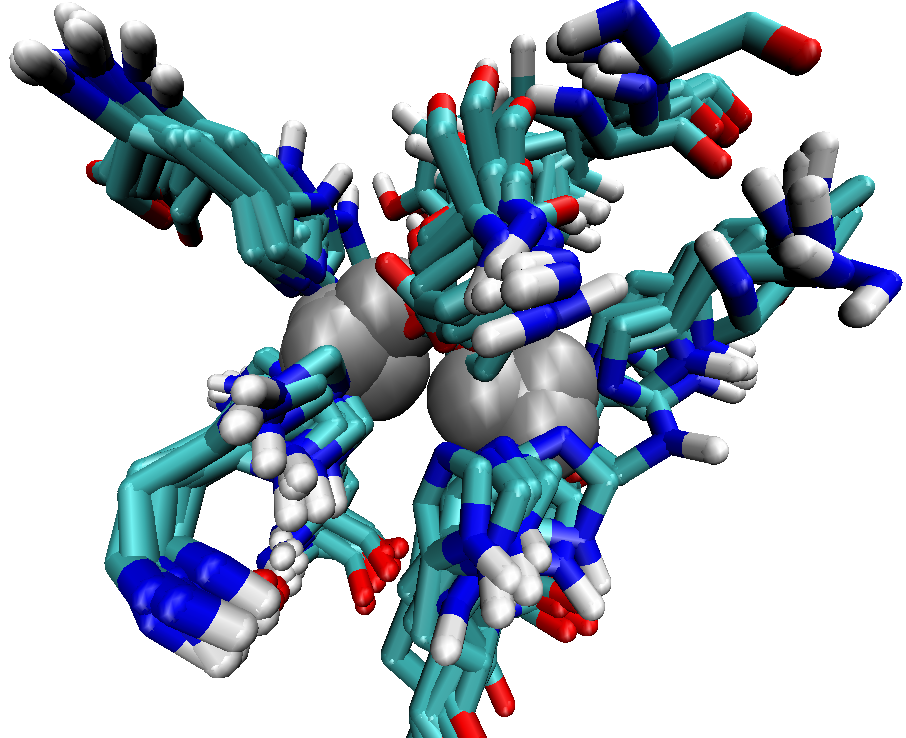

Supplement: Figure S2 — (TIFF) [file pone.0075395.s002.tiff]

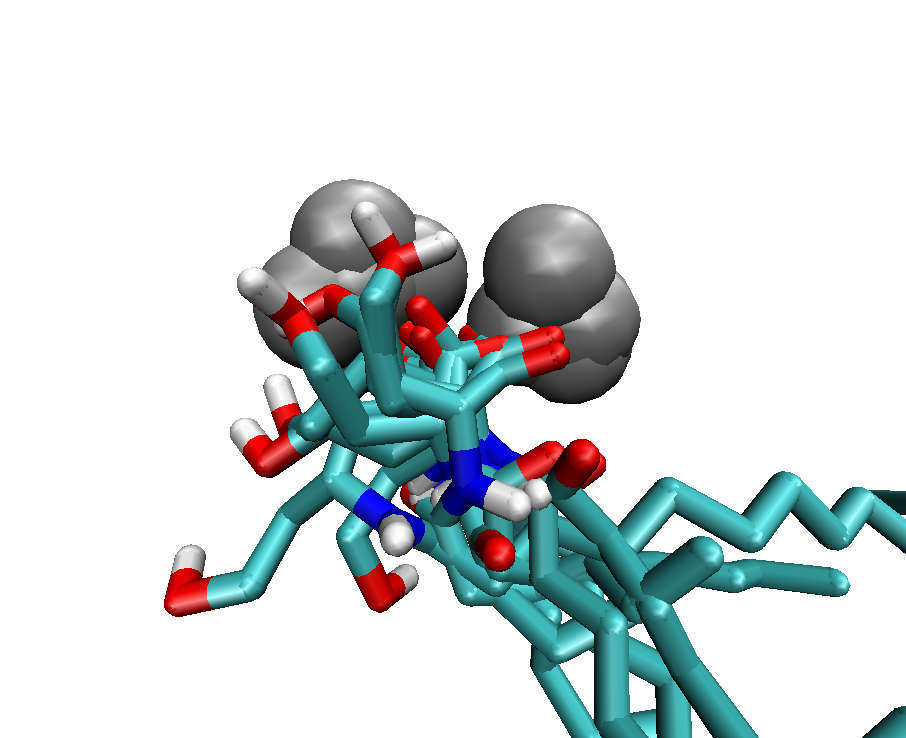

Supplement: Figure S3 — (TIFF) [file pone.0075395.s003.tiff]
